# Higher omega-3 index after dietary inclusion of omega-3 phospholipids versus omega-3 triglycerides in Alaskan Huskies

**DOI:** 10.14202/vetworld.2020.1167-1173

**Published:** 2020-06-22

**Authors:** Lena Burri, Knut Heggen, Andreas Berg Storsve

**Affiliations:** Aker BioMarine Antarctic AS, Lysaker, Norway

**Keywords:** dog, fish oil, krill meal, omega-3 index, phospholipids, triglycerides

## Abstract

**Background and Aim:**

Numerous studies have found benefits of omega-3 polyunsaturated fatty acids (PUFAs), namely, for eicosapentaenoic acid (EPA) and docosahexaenoic acid (DHA) in dogs. The objective of the present study was to assess the efficacy of dietary inclusion of equal amounts of omega-3 FAs in phospholipid (PL) from krill meal to triglyceride structure from fish oil to increase the omega-3 FA profile in red blood cells (RBCs) in dogs.

**Materials and Methods:**

Ten adult Alaskan Huskies of both genders were supplemented with daily 1.7 g EPA and DHA from krill meal for 6 weeks, while another ten dogs received 1.7 g EPA and DHA from fish oil. FA and omega-3 index measurements of the two groups were taken after 0, 3, and 6 weeks for comparison.

**Results:**

It was mainly the EPA levels that increased in the krill meal group (from 1.84% to 4.42%) compared to the fish oil group (from 1.90% to 2.46%) (p<0.001), which drove the group differences in the omega-3 index. This resulted in the krill meal group having a mean omega-3 index increase from 3.9 at baseline to 6.3%, which was significantly greater than the increase from 3.9% to 4.7% observed in the fish oil group (p<0.001). Concomitantly, omega-6 PUFAs, such as arachidonic acid and linoleic acid, were reduced in RBC membranes and the omega-6 to omega-3 ratio was significantly more reduced in the krill meal compared to the fish oil group.

**Conclusion:**

The results showed that krill meal supplementation was associated with a reduction of omega-6 PUFAs, which compensated for the increased omega-3 index, suggesting that PLs are efficient delivery molecules of omega-3 PUFAs.

## Introduction

Eicosapentaenoic acid (EPA; C20:5 n-3) and docosahexaenoic acid (DHA; C22:6 n-3) are two long-chain omega-3 polyunsaturated fatty acids (PUFAs) that are mostly present in cold-water marine organisms such as mackerel, salmon, herring, and krill. These omega-3 PUFAs function in cell membranes as parts of phospholipids (PLs), where they are structural components and increase membrane fluidity. They are also crucial as ligands for transcription factors, thus influencing gene expression, and for an optimal balance with omega-6 PUFAs for the formation of eicosanoids and endocannabinoids [1]. Since they have the ability to replace the pro-inflammatory arachidonic acid (ARA, C20:4 n-6) in membranes and are metabolized into anti-inflammatory signaling molecules, they have the potential to influence the regulation of the inflammatory response. Moreover, they are important for neurological and psychiatric balance and body composition homeostasis [2-4]. In dogs, omega-3 PUFAs have been shown to be beneficial, for example, for cardiovascular system, skin, kidney, joint and brain health, as well as for alleviating symptoms of metabolic disorders and cancer [5]. In addition, top athletes, such as the Alaskan Husky sled dogs participating in long-distance competitions, can profit from omega-3 PUFAs to cope with intense racing conditions and to sustain health and physical performance, as well as to promote quick recovery. Iditarod is perhaps the most extreme endurance exercise performed by any domestic animal, and markers of oxidative stress, inflammation, and skeletal muscle damage were shown to be increased after the race [6]. Nevertheless, a previous study demonstrated that omega-3 PUFAs from krill meal improved inflammation and muscle damage in dogs competing in the 2016 Iditarod dog sled race [7]. In light of these studies, there is a clear interest in increasing EPA and DHA in the diet of all dogs and in particular, for Alaskan Huskies, the animal model of this study.

A typical omega-3 source is fish oil, where the omega-3 PUFAs are bound to triglycerides (TGs), but more recently also products from krill have been included in diets for pets [7,8]. Krill meal is made from Antarctic krill (*Euphausia superba*) to provide a mix of proteins and fat, the latter being characterized by a high content of omega-3 PUFAs bound in PLs [9].

The question of differences in how much of the omega-3 PUFAs are retained in the body in respect to their chemical form from either krill oil or fish oil is addressed in several clinical and pre-clinical studies. The long-term human studies range from 4 to 7 weeks and have analyzed either red blood cell (RBC) membranes in addition to plasma FAs [10,11], only plasma FAs [12,13], or whole blood [14]. The studies were performed in healthy [10,11,14], overweight [12], or normal to slightly elevated total blood cholesterol and/or TG subjects [13].

In short, the study by Maki *et al*. [12] is a randomized, double-blind parallel-arm study that compared a 2 g supplementation of either fish oil, krill oil, or olive oil for 4 weeks. While the received daily amount of EPA was similar in the krill oil and fish oil group (0.216 and 0.212 g, respectively), the DHA quantity was approximately half as much in the krill oil group compared to the fish oil group (0.09 and 0.178 g, respectively). Nevertheless, at the end of the study period, plasma analysis showed that the mean EPA concentrations were higher in the krill oil group compared to the fish oil group, and the mean DHA concentrations were similar in both groups. After dose-adjustment, this results in a 24% higher EPA and DHA plasma increases in the subjects who received omega-3 FAs in PL compared to the subjects who received it in TG form.

The open single-center, randomized parallel study performed by Ulven *et al*. [13] investigated if a 37% lower dose of EPA and DHA provided in PLs (krill oil) compared to omega-3 PUFAs provided in TGs (fish oil) showed equal concentrations of these FAs in plasma after 7 weeks of supplementation. Subjects with normal or slightly increased total blood cholesterol and/or TG levels were randomized into three groups and given either krill oil (0.543 g EPA/DHA), fish oil (0.864 g EPA/DHA), or placebo. The results showed that dietary omega-3 administration led to a similar increase of plasma omega-3 FAs in both the krill and fish oil groups compared to the control group. These findings suggest that a lower dose of EPA and DHA in PL form is required to obtain plasma levels comparable to the TG form of omega-3 supplementation. After adjustment of EPA and DHA levels to the daily dose given, the results from the krill oil group suggest a 45% higher total EPA and DHA plasma concentrations than in the fish oil group at the end of study.

The study by Ramprasath *et al*. [10] was a randomized double-blind crossover trial comparing 3 g of krill oil (0.777 g of EPA/DHA), fish oil (0.663 g EPA/DHA), or corn oil given for 4 weeks to healthy volunteers. Both in plasma and RBCs, EPA and DHA proportions increased significantly in the krill oil group compared to the fish oil group. These results could not be reproduced by Yurko-Mauro *et al*. [11] in a double-blind, randomized, parallel study. Study subjects were supplemented with 0.816 g/d EPA and 0.522 g/d DHA from either fish oil ethyl esters, fish oil, or krill oil for 4 weeks. No significant differences in total plasma and RBC EPA and DHA concentrations were observed at the end of the study.

Finally, an open-label, randomized, cross-over study by Laidlaw *et al*. [14] compared the increase of mean whole blood EPA and DHA concentrations of healthy subject after a 4-week supplementation of concentrated TG (1.1 g of daily EPA/DHA), ethyl ester (0.984 g of EPA/DHA), krill oil (0.24 g of EPA/DHA), or salmon oil (0.4 g of EPA/DHA). The higher doses led to EPA and DHA whole blood concentrations that were significantly higher in the fish oil compared to krill oil groups.

Nowadays, the omega-3 index that represents the EPA and DHA concentrations as a percentage of all erythrocyte FAs has widely been suggested to best represent long-term omega-3 PUFA intakes. Since EPA and DHA become biologically important once they are integrated into membrane PLs, the analysis of RBC membranes provides a convenient way to assess their intake and distribution in other tissues. On the other hand, studies in rodents can more easily rely on direct tissue measurements to assess differences in omega-3 delivery forms. There are three pre-clinical studies that directly compared krill oil with fish oil [15-17] and other studies have looked at TG versus PL forms [18]. Most of these studies have used rats as a model system, but also mice [19], piglets [20], and baboons [21] were included in studies.

So far, no study has investigated the difference in omega-3 PUFA increase in RBCs of dogs when PL versus TG delivery forms were compared. Hence, for the study reported here, we hypothesized that the intake of krill meal by Alaskan Huskies would increase their omega-3 index to a greater extent than would a control diet with omega-3 PUFAs in TG form. The overall purpose of the study was to evaluate the potential of krill meal as a dietary supplement to improve omega-3 PUFA concentrations in dogs.

## Materials and Methods

### Ethical approval

The dogs participating in the study were used to regular blood sample collection because of their dog sled race experience and any discomfort was minimized by sample collection in a known environment at their kennel. Guidelines by the International Animal Ethics Committee were followed, but comparing two different feeds fed to dogs, where no adverse effects of the feeds are expected, does not require approval according to the Norwegian regulation of animal experimentation.

### Animals and blood sampling

A Norwegian dog sled team of 20 Alaskan Huskies with an age range from 2.5 to 5 years (average age of 3.15 years) and a mean body weight of 27.25 kg ranging between 19.1 and 32.3 kg took part in this study during the summer low training period, slightly increasing training in the last weeks of the study. The Alaskan Husky, although not a pure breed, is a top athlete that is specifically bred for long-distance adventures. They are descendants of several breeds, including Alaskan malamutes, Siberian huskies, pointers, setters, Salukis, and Anatolian shepherds. Even though racing Alaskan Huskies only weigh around 25 kg, they are extreme calorie burners with an energy expenditure of up to 12,000 calories (50,000 kJ) per day [22].

Before the treatment period, the dogs were fed for 6 weeks “Labb Adult” (Felleskjøpet Agri SA, Lillestrøm, Norway). The dogs were randomly allocated into two groups, but it was ensured that there were nine males and one female in each group. One group of ten dogs received 8% of a proprietary krill dietary supplement provided by Aker BioMarine Antarctic AS (QRILL™ Pet, Lysaker, Norway) for 6 weeks. The krill meal contained 3 g/kg meal of EPA and DHA, which corresponded to daily intakes of EPA and DHA of around 1.7 g. The second team of ten dogs received a fish oil diet with similar omega-3 PUFA content by the addition of 1.5% fish oil (NorSalmOil, Norsildmel AS, Bergen, Norway), but which was devoid of krill meal. The standard feed for all dogs was based on a commercial diet (Felleskjøpet Agri SA, Lillestrøm, Norway), a nutritionally complete, dry and extruded diet, into which either krill meal or fish oil were added before extrusion. The two diets were formulated to be isonitrogenous and isocaloric and to meet nutrient recommendations [23] for adult dogs (Table-1). The dogs were fed individually every evening, approximately 560 g of dry food. The amount is based on experience on how much food an Alaskan Husky needs in inactive periods. They slept in separate doghouses with *ad libitum* access to drinking water.

**Table-1:** Ingredient composition and calculated content (percent, unless otherwise stated) of the experimental diets.

Ingredient	Fish oil	Krill meal
Krill meal^1^	-	8.21
Salmon protein silage^2,5^	6	6
Fish meal^3^	4	-
Chicken meal^4^	19.91	17.39
Potato starch	17	17
Pea starch	14	14
Rice	21	21
Chicken fat	8.22	8.46
Lard	3	3
Fish oil	1.5	-
Hydrolyzed chicken protein^5^	2	2
Brewer’s yeast	0.75	0.75
Fibrous ingredients	2.84	2.84
Vitamins and minerals	3.56	3.43
Zoo-technical additives	0.23	0.23
Moisture evaporation^5^	−4.01	−4.33
Calculated nutrient contents		
Moisture	7.8	7.6
Dry matter	92.2	92.4
Crude protein	25	25
Crude fat	16	16
Fatty acids		
14:0	0.30	0.32
16:0	3.15	3.20
16:1	0.62	0.61
18:0	1.03	1.02
18:1	5.00	4.97
18:2	2.59	2.56
18:3	0.26	0.25
18:4	0.04	-
20:5 (EPA)	0.13	0.21
22:5	0.02	0.01
22:6 (DHA)	0.17	0.09
EPA + DHA (g/kg)	3.03	3.00
∑ n-3 (g/kg)	5.82	5.59
∑ n-6 (g/kg)	26.31	25.96
n-6/n-3	4.52	4.64
Crude fiber	1.8	1.8
Crude ash	6.5	6.5
NFE^6^	42.9	42.9
Calcium	1.2	1.2
Phosphorous	0.86	0.86
kcal/kg^7^	3764	3768

1 QRILL™ Pet, Aker BioMarine Antarctic AS, Lysaker, Norway,

2 Hordafôr AS, Bekkjarvik, Norway,

3 NorseNAT-LT, Norsildmel AS, Bergen, Norway,

4 Norsk Protein AS, Ingeberg, Norway,

5 Salmon protein silage and hydrolyzed chicken protein have a water content of about 52-62%, most of which evaporates during processing, which were accounted for under moisture evaporation.

6 % NFE=% dry matter - (% crude lipid + % crude protein + % ash + % crude fiber).

7 kcal/kg is the metabolizable energy in dogs [23]. DHA=Docosahexaenoic acid, EPA=Eicosapentaenoic acid, NFE=Nitrogen-free extract

Both groups underwent a physical examination by a veterinarian before blood sample collection at baseline and end of the study about 3-4 h after feeding. During this examination, 6-8 ml of venous blood was collected from the cephalic vein into a vacutainer containing EDTA for plasma collection. RBC and plasma were separated by centrifugation at 3000 rpm for 15 min at room temperature and kept on dry ice until stored at −80°C. The plasma was used for the analysis of choline and its metabolites and the results are described in a separate publication [24]. RBCs were analyzed as described previously at Omegametrix GmbH (Martinsried, Germany) [25]. In short, FA methyl esters from RBCs were identified by gas chromatography (GC2010, Shimadzu, Duisburg, Germany) equipped with a SP2560 100-m column (Supelco, Bellefonte, PA, USA) using hydrogen gas as a carrier by comparison with a known standard (GLC-727; Nuchek Prep, Elysian, MN, USA). Omega-3 index was given as EPA + DHA in RBCs expressed as a percentage of the total identified FAs.

### Statistical analysis

Mixed-design two-way ANOVA was employed to test for overall and interaction effects of time point (baseline, 3 weeks, and 6 weeks; within subject factor) and supplement type (krill meal and fish oil; between subject factors) on RBC membrane FA composition. In cases where significant time point × supplement type interaction effects were found, independent sample t-tests were carried out to compare group differences in FA magnitude of change. Alpha was set at 0.05.

## Results

The samples were compared from a group of ten dogs (nine males and one female) receiving 8% krill meal to a control group (nine males and one female) not receiving krill meal, but the same amount of omega-3 PUFAs from fish oil. The proximate composition of dry food given to the animals is presented in Table-1. No diet-related differences were noted for food consumption or body weight over the study period.

### Omega-3 index

**Figure-1:**
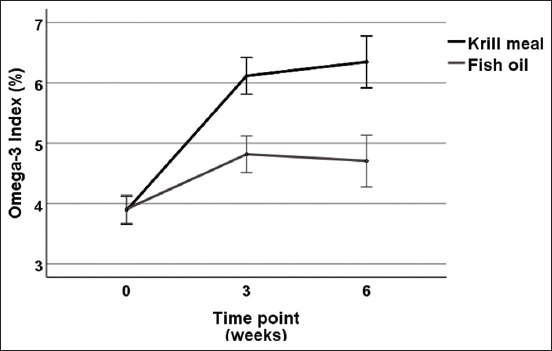
Mean (±95% confidence interval) omega-3 index values (%) across the study period by supplement type.

Figure-1 shows supplement-dependent changes in omega-3 index, with both groups experiencing an increase in the omega-3 index across the study period. A two-factor mixed-design ANOVA revealed a significant effect of time point (p<0.001) and supplement type (p<0.001), as well as a significant time point × supplement type interaction effect (p<0.001). Inspection of Figure-1 suggests that this interaction effect is primarily due to the krill meal group showing a more pronounced increase in omega-3 index levels following supplementation, when compared to the fish oil group. The krill meal group had a mean increase from baseline from 3.9% to 6.3%, and this was significantly greater than the increase from 3.9% to 4.7% observed in the fish oil group (p<0.001).

### Individual FAs

Table-2 shows RBC membrane FA composition at baseline (0 weeks), study mid-point (3 weeks) and study end (6 weeks) for the krill meal group and the fish oil group. In general, an increase in omega-3 FAs and a decrease in omega-6 FAs were observed in both groups across the study period. However, for EPA (C20:5 n-3) there was a significant time point × supplement type interaction effect (p<0.001), and this was due to a more pronounced increase in EPA levels in the krill meal group (from 1.84% at baseline to 4.42% at study end) compared with the fish oil group (from 1.90% to 2.46%) (p<0.001), suggesting that this FA is the key driver of group differences in the omega-3 index. For DHA (C22:6 n-3), there was a significant time point × supplement type interaction effect (p<0.001), due to the fish oil group being the only group to show a significant increase in DHA levels from baseline (2.01%) to study end (2.24%) (p<0.001). Furthermore, for docosapentaenoic acid (DPA; C22:5 n-3), there was a significant time point × supplement type interaction effect (p<0.001) due to the krill meal group showing an increase (from 1.00% to 1.15%) and the fish oil group showing a decrease (from 0.95% to 0.79%) across the study period. Both groups showed reductions in the abundant omega-6 PUFA, linoleic acid (C18:2 n-6), but there was a significant time point × supplement type interaction effect (p<0.001), mainly driven by a larger reduction in the krill meal group (from 22.0% to 19.2%) than the fish oil group (from 22.2% to 20.5%) from baseline to study end. However, this group difference in magnitude of change was not significant (p=0.12). Furthermore, there was a significant main effect of time point on the omega-6 PUFA ARA (C20:4 n-6) due to a decrease in this FA across the study period in both groups (from 17.5% to 16.1% in the krill meal group and from 17.2% to 15.7% in the fish oil group), but no significant effect of supplement type (p=0.69) and no significant interaction effect (p=0.69). It should be pointed out, however, that within-group paired samples t-tests revealed that the reduction in ARA across the study period was significant in the krill meal group (p=0.019), but not the fish oil group (p=0.079).

**Table-2:** Mean (±SE) fatty acid composition of RBC membranes (%) across the study period by supplement type (K, krill meal, and F, fish oil).

Fatty acid (%)		Diet	0 week	3 weeks	6 weeks
∑Trans		K	0.61±0.02	0.56±0.01	0.56±0.02
		F	0.64±0.01	0.54±0.02	0.55±0.02
∑Saturated		K	38.18±0.22	39.76±0.21	39.60±0.25
		F	37.88±0.32	38.56±0.22	39.75±0.26
∑Monounsaturated		K	13.87±0.32	13.32±0.22	14.33±0.45
		F	14.22±0.45	13.54±0.29	15.22±0.40
Polyunsaturated	18:3 n-3 (ALA)	K	0.39±0.02	0.27±0.01	0.33±0.02
		F	0.44 ±0.03	0.37±0.02	0.44±0.03
	20:5 n-3 (EPA)	K*	1.84±0.08	3.88±0.15	4.42±0.25
		F	1.90±0.08	2.28±0.11	2.46±0.14
	22:5 n-3 (DPA)	K*	1.00±0.08	1.22±0.07	1.15±0.05
		F	0.95±0.03	0.85±0.04	0.79±0.02
	22:6 n-3 (DHA)	K	2.05±0.07	2.23±0.07	1.93±0.07
		F*	2.01±0.08	2.53±0.10	2.24±0.08
	18:2 n-6 (LA)	K	22.00±0.42	18.96±0.59	19.26±0.42
		F	22.27±0.61	21.31±0.43	20.49±0.51
	20:4 n-6 (ARA)	K	17.52±0.42	17.43±0.39	16.10±0.53
		F	17.17±0.53	17.56±0.35	15.70±0.51
∑n-3		K*	5.29±0.11	7.61±0.15	7.82±0.24
		F	5.30±0.16	6.04±0.12	5.94±0.20
∑n-6		K	42.05±0.30	38.75±0.30	37.68±0.45
		F	41.95±0.21	41.31±0.22	38.54±0.38
n-6/n-3		K^#^	7.99±0.20	5.11±0.11	4.87±0.20
		F	7.99±0.28	6.86±0.14	6.56±0.23

*^#^Significant between-group difference in magnitude of change from baseline (week 0) to study end (week 6)(p<0.05)^*^ denotes that the respective group showed a significantly more pronounced increase as compared to the other group, while ^#^denotes that the respective group showed a significantly more pronounced decrease as compared to the other group. ALA=Alpha-linolenic acid, DPA=Docosapentaenoic acid, DHA=Docosahexaenoic acid, EPA=Eicosapentaenoic acid, ARA=Arachidonic acid, LA=Linoleic acid, RBC=Red blood cells

### Sum of FAs

As shown in Table-2, there was a decrease in RBC membrane trans FAs (from 0.61% to 0.56% in the krill meal group and 0.64% to 0.55% in the fish oil group) and increases in saturated (from 38.2% to 39.6% in the krill meal group and 37.9 to 39.8% in the fish oil group) and monounsaturated (from 13.9% to 14.3% in the krill meal group and 14.2 to 13.5% in the fish oil group) FAs across the study period. ANOVA revealed significant main effects of time point for trans FAs (p<0.001), saturated FAs (p<0.001), and monounsaturated FAs (p=0.003), but no significant main effects for supplement type (all p>0.05). Furthermore, although the sum of PUFAs was reduced for both groups across the study period, a general pattern of greater increases in omega-3 FAs and more pronounced decreases in omega-6 FAs could be observed in the krill meal relative to the fish oil group. Thus, for the omega-6/omega-3 ratio ANOVA revealed a significant time point × supplement type interaction effect (p<0.001) due to the krill meal group showing a more pronounced decrease (from 7.99 at baseline to 4.87 at study end ) than the fish oil group (from 7.99 at baseline to 6.56 at study end) (p<0.001).

## Discussion

This study was conducted to quantify the uptake of similar doses of EPA/DHA present in different chemical forms (PLs vs. TGs) into RBC membranes of dogs. Due to ethical reasons, no whole body or tissue analysis could be performed, and the omega-3 index measured in RBCs was chosen as a non-invasive marker for tissue PUFA levels. This is possible because the erythrocyte membrane represents a prototype cell membrane, since the FA composition correlates to other tissue membranes and it is easily available. Measurements in RBCs have the advantage to represent long-term omega-3 intake with less variability than plasma measurements [26].

The influence of the different delivery molecules became evident already after 3 weeks of feeding, and at study end there was a 62% increase in the omega-3 index in the krill meal group (PL), whereas the TG form of the fish oil group was associated with a 21% increase. A limitation of the study was that the individual EPA and DHA amounts given at the start of the feeding period differed between the groups and only their sum was the same. EPA and DHA might have different kinetics, which were not accounted for in the study.

EPA/DHA can only increase in erythrocyte membranes, when the relative amount of other FAs decreases in parallel and thereby also their bioactive derivatives. This study found that there was a compensatory decrease in omega-6 PUFAs, i.e., ARA decreased by 8.1% in the krill meal group, which contributed to a reduced omega-6 to omega-3 PUFA ratio. The end result is therefore a combination of the effects of increased EPA/DHA levels and a shift to reduced production of ARA-derived pro-inflammatory mediators moving toward an anti-inflammatory immune state. Lower omega-6 and higher omega-3 levels are of functional relevance to the health of dogs and it is therefore of interest to increase their omega-3 index. Relevant for a future study could also be the analysis of downstream metabolites, such as the two endocannabinoids derived from ARA, anandamide and ­2-arachidonoylglycerol or the acylethanolamide endocannabinoid-like mediators, i.e., EPA-ethanolamide, DHA-ethanolamide, oleoylethanolamide, and palmitoylethanolamide, which are the ethanolamides of EPA, DHA, oleic acid, and palmitic acid, respectively. This would give insights into correlations of changes in membrane FA composition and their metabolite plasma concentrations, which is important since endocannabinoids can have regulatory effects on appetite, food intake, fat accumulation and insulin sensitivity [27]. Another interesting focus area for a potential follow-up study could be the specialized ­pro-resolving mediators (SPM-lipoxins, resolvins, protectins, and maresins) that are produced by enzymatic conversion of essential FAs, including omega-3 PUFAs. Inasmuch, DHA- and EPA-derived SPMs may help in the reduction of vascular and systemic inflammation [28]. Hence, increasing dietary omega-3 intake may have wide health benefits that are not limited to omega-3 PUFAs directly [1].

Noteworthy is a 6-week study that analyzed whole body omega-3 PUFA deposition in rats [29]. The results showed that around 13% more omega-3 PUFAs accumulated over all the body tissues when originating from krill oil compared to fish oil. They also found that more than 90% of EPA and more than 60% of DHA were b-oxidized in both diet groups. The authors suggested that it is the degradation and deposition, but not the absorption of PUFAs that is affected by the oil source (krill or fish). Other evidence further suggests that after digestion and absorption, the PUFAs from PLs go a different route than those from TGs and end up in different blood pools (free FAs, outer vs. inner layer of chylomicrons, and different lipoprotein particles) [20]. This might influence the fate of PUFAs in the body and could explain the findings of this study.

Ghasemifard *et al* [29]. also highlighted that when looking at the whole body in rats, a higher PUFA proportion of around 10% was deposited in female compared to male rats, a finding found to be independent of diet. This is a limitation in the present study, where out of the ten dogs only one was female in each group, so no gender-specific observations could be made. Since females have higher omega-3 PUFA requirements because of their importance for the offspring during pregnancy and lactation [30-32], it would be of interest to see if female dogs have a higher ability to not only retain EPA and DHA but also produce more long-chain from short-chain omega-3 PUFAs as seen in humans [33-35].

## Conclusion

Six weeks of 8% krill meal supplementation in dry, extruded feed is an efficient mean to increase the omega-3 index of dogs. This is of importance since an increased omega-3 PUFA tissue content will affect many biochemical and physiological functions in dogs that can result in health benefits e.g. skin, heart, liver, and the immune system.

## Authors’ Contributions

KH and LB designed the study protocol and were involved with sample collection, LB wrote the paper and ABS performed the data analysis and was involved with drafting of the manuscript. All authors revised, read, and approved the final manuscript.
